# Identifying Factors for Low-Risk Participation in Alternative Cardiac Rehabilitation Models for Patients with Coronary Heart Disease Using MI'S SCOREPAD

**DOI:** 10.1155/2023/7230325

**Published:** 2023-09-08

**Authors:** Eric J. Brandt, Joshua Garfein, Chih-Wen Pai, Joseph Bryant, Eva Kline-Rogers, Samantha Fink, Melvyn Rubenfire

**Affiliations:** ^1^Division of Cardiovascular Medicine, Department of Internal Medicine, University of Michigan, Ann Arbor, MI, USA; ^2^Institute for Healthcare Policy and Innovation, University of Michigan, Ann Arbor, MI, USA

## Abstract

**Introduction:**

Although a recent joint society scientific statement (the American Association of Cardiovascular Pulmonary Rehabilitation, the American Heart Association, and the American College of Cardiology) suggests home-based cardiac rehab (CR) is appropriate for low- and moderate-risk patients, there are no paradigms to define such individuals with coronary heart disease.

**Methods:**

We reviewed a decade of data from all patients with coronary heart disease enrolled in a single CR center (University of Michigan) to identify the prevalence of low-risk factors, which may inform on consideration for participation in alternative models of CR. Low-risk factors included not having any of the following: metabolic syndrome, presence of implantable cardioverter defibrillator or permanent pacemaker, active smoking, prior stroke, congestive heart failure, obesity, advanced renal disease, poor exercise capacity, peripheral arterial disease, angina, or clinical depression (MI'S SCOREPAD). We report on the proportion of participants with these risk factors and the proportion with all of these low-risk factors.

**Results:**

The mean age of CR participants (*n* = 1984) was 63 years; 25% were women, and 82% were non-Hispanic White. The mean number of low-risk factors was 8.5, which was similar in the 2011-2012 and 2018-2019 cohorts (8.5 vs. 8.3, respectively, *P* = 0.08). Additionally, 9.3% of the 2011-2012 cohort and 7.6% of the 2018-2019 cohort had all 11 of the low-risk factors.

**Conclusion:**

In this observational study, we provide a first paradigm of identifying factors among coronary heart disease patients that may be considered low-risk and likely high-gain for participation in alternative models of CR. Further work is needed to track clinical outcomes in patients with these factors to determine thresholds for enrolling participants in alternative forms of CR.

## 1. Introduction

Cardiac rehabilitation (CR) is effective for secondary prevention following a major adverse cardiovascular event [1, 2]. CR has a class I indication with evidence that comprehensive medically supervised risk reduction strategies lead to improved compliance with healthy lifestyles and evidence-based therapies, quality of life, cardiorespiratory fitness, and exercise tolerance, quicker return to work, and decreases in angina, subsequent hospitalizations, and mortality [3]. CR is entering an era wherein alternative forms of CR, including abbreviated, home-based, or hybrid CR, are being increasingly considered, especially for some patients with coronary heart disease (CHD).

A recent joint Scientific Statement from the American Association of Cardiovascular Pulmonary Rehabilitation (AACVPR), the American Heart Association (AHA), and the American College of Cardiology (ACC) states that home-based CR “may be a reasonable option for selected clinically stable low- to moderate-risk patients” [4] However, no definition for low or moderate risk is provided in this scientific statement. There are definitions in the AACVPR Guidelines for CR programs (6^th^ Edition) for low or moderate risk factors for participation in CR's exercise component. However, there remain no paradigms to define individuals with CHD who may be low enough risk based on exercise and nonexercise factors and possibly more suitable for alternative nontraditional CR models (i.e., abbreviated, home-based, or hybrid CR).

Thus, we reviewed a decade of data from all patients with CHD enrolled in a single CR center to identify the prevalence of low-risk factors that may be considered for suitable participation in alternative models of CR. We hypothesized that commonly recognized low-risk factors would be common in the CR population. Furthermore, since comorbidities have shifted over time in the general population, we hypothesized that the prevalence of these risk factors would differ in the first and last two years of the cohort.

## 2. Methods

### 2.1. Data

This prospective observational study followed the Strengthening the Reporting of Observational Studies in Epidemiology reporting guidelines. Our study consisted of patients enrolled in the University of Michigan CR program from 1/1/2011–2/29/2020 whose indication for CR was CHD-related (*n* = 1984). This study was approved by the University of Michigan IRB. Consent to participate in CR and for data to be used for research purposes were obtained from all participants at the time of CR enrollment (IRB#: HUM00045929).

Self-reported patient characteristics were collected from standardized health questionnaires and at intake via exercise physiologists, including age, sex, race/ethnicity, physical activity level, smoking status, and history of peripheral arterial disease. Stroke, heart failure, and presence of cardiac pacemaker or implantable cardioverter defibrillator history were derived from past diagnostic codes. Angina was based on indication for referral as documented by the exercise physiologist. Psychological distress was evaluated using the Brief Symptom Inventory (BSI), consisting of 53 items and covering nine symptom dimensions [5]. Adult nonpatients were used as the reference group to convert raw scores to *T*-scores. Depression was defined as a *T*-score of ≥63 (90^th^ percentile) [6]. Ejection fraction was obtained from the ventriculogram or, if not available, the most recent echocardiogram or nuclear study. Definitions for major CHD risk factors and the criteria for the metabolic syndrome were based upon those of the American Heart Association [7]. For determining the metabolic syndrome, we used laboratory data (fasting glucose and lipid profiles) from the date closest to baseline CR evaluation. Clinical exercise physiologists measured blood pressure, body weight, and waist circumference at CR entry. Cardiorespiratory fitness was measured at CR entry by peak oxygen consumption (VO_2peak_) and evaluated using electronic/motorized treadmill test (Ultima CPX metabolic stress test system, MCG Diagnostics). Patients were tested until requesting to stop, general/leg fatigue, clinical decision to terminate, or maximal effort. Patients were not asked to discontinue medications before measurement.

### 2.2. Outcome

The primary outcome was the proportion of CR participants who were low-risk and therefore could likely qualify for abbreviated, home, or hybrid CR participation. We were able to identify 11 commonly measured factors in CR and defined low risk as having all 11 of these low-risk factors.

The factors included five factors that the AACVPR defines as low-risk factors for participating in the exercise component of CR, including peak aerobic capacity ≥7 METS (METS = VO_2peak_/3.5), no heart failure (including ejection fraction (EF) ≥ 50%), no angina, and no clinical depression [8, 9]. Presence of cardiac pacemaker or implantable cardioverter defibrillator was available and used as a substitute for risk for complex dysrhythmia. Notably, the AACVPR also identified complex dysrhythmia and abnormal hemodynamics during exercise testing and recovery as risk factors, but these data were not available for this study.

We expanded on the AACVPR risk factors by including six additional factors that do not increase the risk of participating in the exercise component of CR but require major lifestyle changes that may not be equally addressed from alternative CR approaches or are major cardiovascular risk factors. These additional low-risk factors included current nonsmoking status, class I obesity or less (BMI ≤ 35 kg/m^2^), no metabolic syndrome, no peripheral arterial disease, no prior stroke, no advanced renal disease (eGFR ≥ 45 ml/min/m^2^), and no depression. These risk factors can be recalled using the acronym MI'S SCOREPAD (see Table 1 for review of risk factor definitions).

### 2.3. Statistical Analysis

Among the entire study cohort, the extent of missing data for the relevant factors ranged from 0% to less than 10%, except for exercise capacity measure (36%). Thus, we performed multiple imputations with 100 imputed datasets, which were combined for estimates of the individual factors and corresponding comparisons. Nonimputed results are reported in Supplementary Table 1. Comparisons were made using chi-squared tests, Fisher's exact tests, and Wilcoxon rank-sum tests. We compared the prevalence of factors in the earliest (2011-2012) and most contemporary (2018-2019) cohorts. All analyses were performed using SAS v9.4 (SAS Institute, Inc.). A 2-tailed *P* < 0.05 was used to indicate statistical significance.

## 3. Results

The mean age of CR participants (*n* = 1984) was 63 years; 25% were women, and 82% were non-Hispanic White (Table 2). The 2018-2019 cohort was older than the 2011-2012 cohort (*P* < .001).

Proportions of participants with factors associated with a low-risk for participation in abbreviated/home/hybrid CR are provided in Table 3. The mean number of low-risk factors was 8.5. Additionally, 7.3% of the entire cohort had all 11 of the low-risk factors. Mean risk factors and number with all 11 risk factors were similar in the 2011-2012 and 2018-2019 cohorts (8.5 vs. 8.3, *P* = 0.08, and 9.3% vs. 7.6%, *P* = 0.70, respectively). However, compared to the 2011-2012 cohort, the 2018-2019 cohort was more likely to have a history of congestive heart failure (*P* = 0.002) and advanced renal disease (*P* = 0.03).

Among the five AACVPR-driven guideline-based factors, the median number of low-risk factor was 4 and mean was 3.5 (Table 3). There were 12.5% of the cohort with all five of the AACVPR low-risk factors, which was similar in the 2011-2012 (13.0%) and 2018-2019 (14.0%) cohorts (*P* = 0.67).

Values without multiple imputation are reported in Supplementary Table 1.

## 4. Discussion

In this observational study, we provide a first paradigm of identifying factors among CHD patients that may be considered low-risk and likely high-gain for participation in alternative models of CR (i.e., abbreviated, home-based, or hybrid CR). These factors are overall common with most having at least 8 low-risk factors. Our findings are relevant to ongoing decisions for the future of CR and how CR can better serve the needs of current patients with CHD.

Although paradigms such as home-based CR are being used clinically [10], there is no guideline-chosen paradigm to define level of risk and aid in deciding which patients are best qualified for different lengths and types of CR. As new types of CR evolve, there should be ongoing consideration of which factors inform the preferred modality. It is important to understand this because alternative forms of CR, such as home-based CR, may be just as, if not more, cost-effective than center-based options, but patients should be chosen wisely to ensure safety [11–13].

We have provided a first paradigm of factors based on currently recognized clinical risk factors to determine which patients may be low-risk for alternatives to inperson CR. Notably, 7.3% of participants had all 11 factors from the MI'S SCOREPAD and 12.5% of participants has all five AACVPR guideline-driven criteria. Our analysis does not determine an appropriate cut point for alternative CR models. This research is direly needed. What our work does do is lend a view into a potential population for these future CR strategies.

### 4.1. Limitations

First, the study utilized medical records to identify relevant data, some of which were not originally collected for research purposes. Second, there were a large number of missing data and repeat analysis after multiple imputation did not confirm all findings. Third, our patient sample was predominantly White at a major academic medical center, which limits generalizability.

## 5. Conclusion

This study introduces a novel framework of factors to consider for determining whether CHD patients are at low-risk for alternative models of CR. Given the urgent need for enhanced approaches to secondary CHD prevention [14], additional study is warranted to optimize CR programs to increase accessibility and promote cardiometabolic health [15]. Our findings can provide a basis for future studies that seek to clearly define low and moderate risk for participation in home-based CR and other CR models based on recent joint-society scientific statements.

## Figures and Tables

**Table 1:** MI'S SCOREPAD variables and definitions.

Variable	Definition
No metabolic syndrome	<3 risk factors (of 5): (1) Fasting blood sugar ≥100 mg/dL or prior diagnosis of type 2 diabetes mellitus (2) Triglycerides ≥150 mg/dL or known treatment for hypertriglyceridemia (3) HDL <40 mg/dL for men or <50 mg/dL for women or known treatment for low HDL (4) Systolic blood pressure ≥130 mmHg or diastolic blood pressure ≥85 mmHg or treatment of previously diagnosed hypertension (5) Waistline >40 inches (>102 cm) for men or >35 inches (>88 cm) for women
No implantable cardioverter defibrillator/cardiac pacemaker placement	Indicated never had placed either of these cardiac devices
Current nonsmoker	No patient-reported history of active tobacco smoking
No prior stroke	No patient-reported history of prior stroke
No congestive heart failure	No patient-reported history of clinical heart failure and EF ≥ 50%
No severe obesity	BMI ≤ 35 kg/m^2^
No advanced renal disease	eGFR ≥ 45 L/min/1/. 73m^2^
Good exercise capacity	Peak aerobic capacity ≥7 METS
No peripheral arterial disease	No patient-reported history of peripheral arterial disease
No angina	No patient report of angina at cardiac rehab intake
No depression	Brief Symptom Inventory T-score <63

**Table 2:** Characteristics of cardiac rehabilitation study population, 2011-2020, and at the beginning and end of the study period.

Variable	Total *N* = 1984	2011-2012 *n* = 341	2018-2019 *n* = 516	*s* value^∗^
Age^†^	63 (11)	62 (11)	64 (12)	<.001
Age group^‡^	—	—	—	.01
<40	49 (2.5)	12 (3.5)	16 (3.1)	—
40-49	171 (8.6)	34 (10.0)	45 (8.7)	—
50-59	471 (23.7)	87 (25.5)	110 (21.3)	—
60-69	716 (36.1)	128 (37.5)	166 (32.2)	—
70-79	425 (21.4)	63 (18.5)	122 (23.6)	—
≥80	152 (7.7)	17 (5.0)	57 (11.0)	—
Male^‡^	1486 (74.9)	262 (76.8)	396 (76.7)	.99
Race/ethnicity^‡^	—	—	—	.11
Non-Hispanic Asian	120 (6.0)	16 (4.7)	38 (7.4)	—
Non-Hispanic Black	117 (5.9)	23 (6.7)	27 (5.2)	—
Hispanic	33 (1.7)	3 (0.9)	15 (2.9)	—
Non-Hispanic White	1624 (81.9)	285 (83.6)	412 (79.8)	—
Other	90 (4.5)	14 (4.1)	24 (4.7)	—

^∗^ *P* value from Wilcoxon rank-sum test, chi-squared test, and Fisher exact test (e.g., 2010-2011 vs. 2018-2019). ^†^Data presented as mean (SD). ^‡^Data presented as n (%).

**Table 3:** Low-risk factors to consider for favorable participant in alternative forms of cardiac rehabilitation based on 100 imputed datasets.

Variable	Total *N* = 1984	2011-2012 *n* = 341	2018-2019 *n* = 516	*P* value^∗^
—	%	%	%	—
No metabolic syndrome	43.7%	44.9%	43.6%	0.71
Good exercise capacity^†^	20.4%	19.4%	23.4%	0.18
No prior stroke	74.6%	76.5%	74.2%	0.44
No PAD	94.1%	94.7%	93.9%	0.64
No CHF	61.1%	68.5%	57.6%	0.002
No ICD/cardiac pacemaker placement	92.6%	91.5%	91.6%	0.78
No stable angina	95.2%	96.8%	95.7%	0.47
Current nonsmoker	94.7%	94.1%	93.6%	0.77
No severe obesity (BMI ≤35kg/m^2^)	83.1%	85.0%	83.5%	0.57
No advanced renal disease (eGFR ≥45 ml/min/m^2^)	93.1%	95.9%	92.2%	0.03
No depression	84.7%	85.9%	82.9%	0.26
Number of factors	—	—	—	0.70
1	0.1%	0.0%	0.2%	
2	0.1%	0.0%	0.2%	
3	0.1%	0.0%	0.2%	
4	0.8%	0.4%	1.0%	
5	3.0%	2.6%	3.0%	
6	7.3%	5.1%	7.0%	
7	16.0%	17.0%	17.0%	
8	23.6%	22.6%	24.6%	
9	25.0%	24.4%	23.2%	
10	16.9%	18.7%	16.2%	
11	7.3%	9.3%	7.6%	
All Factors	—	—	—	—
Median (IQR) number of factors	9 (7.6, 10.0)	9 (7.6, 10.0)	8 (7.0, 9.0)	0.13
Mean number of factors	8.5	8.5	8.3	0.08
AACVPR guideline factors only^‡^	—	—	—	—
Median (IQR) number of factors	4 (3, 4)	4 (3, 4)	4 (3, 4)	0.07
Mean number of factors	3.5	3.6	3.5	0.11

Footnote: BMI = body mass index; CHF = congestive heart failure; ICD = implantable cardioverter defibrillator; IQR = interquartile range; PAD = peripheral artery disease; SD = standard deviation. ^∗^*P* value from *χ*^2^ tests. †Defined as able to achieve ≥7 METS. ^‡^AACVPR factors included peak aerobic capacity ≥7 METS (METS = VO_2peak_/3.5), no heart failure (including ejection fraction (EF) ≥ 50%), no angina, no clinical depression, and presence of cardiac pacemaker or implantable cardioverter defibrillator.

## Data Availability

The datasets generated during and/or analyzed during the current study are not publicly available due to lack of approval for data use in this manner. We are agreeable to confirming study details necessary to understand our results.

## Additional Points

*Key Summary Points*. Why carry out this study? (i) It has been recommended to consider low-risk patients for alternative forms of cardiac rehab (such as at-home rehab). However, no clear paradigms exist to determine definitively who meets threshold for low-risk. (ii) We hypothesized that factors to deem a patient low risk would be common among a cardiac rehab population. Thus, we identified 11 commonly recognized factors that could be considered low risk and calculated their prevalence from 10 years of cardiac rehab data. What was learned from this study? (i) The main outcome of our study was the prevalence of low-risk factors among our cardiac rehab population. The mean number of low-risk factors was 8.5. Additionally, 7.3% had all of the low-risk factors. (i) We learned that low-risk factors are common among cardiac rehab patients. Further work needs to be done to correlate number of factors with cardiovascular outcomes.

## Supplementary Materials

Supplementary Materials Supplementary Table 1: low-risk factors to consider for favorable participant in alternative forms of cardiac rehabilitation—this table shows the results of the nonimputed data.
