# Supplementary material for: Identifying Factors for Low-Risk Participation in Alternative Cardiac Rehabilitation Models for Patients with Coronary Heart Disease Using MI'S SCOREPAD
Source: Cardiovasc Ther. 2023 Sep 8;2023:7230325. doi: 10.1155/2023/7230325 (PMC10504043; doi:10.1155/2023/7230325)
Supplement: Supplementary Materials — Supplementary Table 1: low-risk factors to consider for favorable participant in alternative forms of cardiac rehabilitation—this table shows the results of the nonimputed data. [file 7230325.f1.docx]

| **Supplementary Table 1.** Low-Risk Factors to Consider for Favorable Participant in Alternative Forms of Cardiac Rehabilitation | | | | | | | |
| --- | --- | --- | --- | --- | --- | --- | --- |
| **Variable** | **Total**  **N = 1984** | | **2011-2012**  **n = 341** | | **2018-2019**  **n = 516** | | **P Value*** |
| - | Number with Data Available | N (%) | Number with Data Available | N (%) | Number with Data Available | N (%) | - |
| No metabolic syndrome | 1845 | 775 (42.0%) | 324 | 141 (43.5%) | 460 | 189 (41.1%) | 0.51 |
| Good exercise capacity† | 1268 | 299 (23.6%) | 286 | 54 (18.9%) | 210 | 79 (37.6%) | <0.001 |
| No prior stroke | 1797 | 1316 (73.2%) | 290 | 216 (74.5%) | 474 | 345 (72.8%) | 0.61 |
| No PAD | 1941 | 1829 (94.2%) | 329 | 312 (94.8%) | 511 | 480 (93.9%) | 0.65 |
| No CHF | 1977 | 1237 (62.6%) | 339 | 237 (69.9%) | 516 | 306 (59.3%) | 0.002 |
| No ICD/cardiac pacemaker placement | 1807 | 1680 (93.0%) | 292 | 269 (92.1%) | 478 | 439 (91.8%) | 1.00 |
| No stable angina | 1984 | 1889 (95.2%) | 341 | 330 (96.8%) | 516 | 494 (95.7%) | 0.47 |
| Current non-smoker | 1984 | 1878 (94.7%) | 341 | 321 (94.1%) | 516 | 483 (93.6%) | 0.77 |
| BMI ≤35kg/m^2^ | 1984 | 1648 (83.1%) | 341 | 290 (85.0%) | 516 | 431 (83.5%) | 0.57 |
| No advanced renal disease (eGFR >45 ml/min/m^2^) | 1847 | 1717 (93.0%) | 309 | 297 (96.1%) | 484 | 445 (91.9%) | 0.03 |
| No depression | 1923 | 1631 (84.8%) | 333 | 286 (85.9%) | 484 | 402 (83.1%) | 0.28 |
| Number of factors | 1019 | - | 210 | - | 178 | - | 0.55 |
| 4 | - | 6 (0.6%) | - | 0 (0.0%) | - | 2 (1.1%) | - |
| 5 | - | 20 (2.0%) | - | 6 (2.9%) | - | 1 (0.6%) | - |
| 6 | - | 57 (5.6%) | - | 10 (4.8%) | - | 3 (1.7%) | - |
| 7 | - | 127 (12.5%) | - | 32 (15.2%) | - | 19 (10.7%) | - |
| 8 | - | 249 (24.4%) | - | 51 (24.3%) | - | 48 (27.0%) | - |
| 9 | - | 273 (26.8%) | - | 54 (25.7%) | - | 41 (23.0%) | - |
| 10 | - | 200 (19.6%) | - | 38 (18.1%) | - | 40 (22.5%) | - |
| 11 | - | 87 (8.5%) | - | 19 (9.0%) | - | 24 (13.5%) | - |
| All Factors | - | - | - | - | - | - | - |
| Median (IQR) Number of Factors | 1019 | 9 (8, 10) | 210 | 9 (8, 10) | 178 | 9 (8, 10) | 0.15 |
| Mean Number of Factors (SD) | 1019 | 8.6 (1.4) | 210 | 8.6 (1.4) | 178 | 8.9 (1.4) | 0.03 |
| AACVPR Guideline Factors only ‡ | - | - | - | - | - | - | - |
| Median (IQR) Number of Factors | 1122 | 4 (3, 4) | 238 | 4 (3, 4) | 190 | 4 (3, 4) | 0.005 |
| Mean Number of Factors | 1122 | 3.7 (0.9) | 238 | 3.6 (0.8) | 190 | 3.9 (0.9) | <0.001 |

Footnote: BMI = body mass index; CHF = congestive heart failure; ICD=Implantable cardioverter defibrillator; IQR = interquartile range; PAD = peripheral artery disease; SD = standard deviation

­* P-value from Fisher’s exact tests.

† Defined as able to achieve ≥7 METS

‡ AACVPR factors included peak aerobic capacity ≥7 METS (METS = VO_2peak_/3.5), no heart failure (including ejection fraction (EF) ≥ 50%), no angina, no clinical depression, and presence of cardiac pacemaker or implantable cardioverter defibrillator
